# Does Thinking in Opposites in Order to Think Differently Improve Creativity?

**DOI:** 10.3390/jintelligence11050085

**Published:** 2023-04-30

**Authors:** Ivana Bianchi, Erika Branchini

**Affiliations:** 1 Department of Humanities (Section Philosophy and Human Sciences), University of Macerata, Via Garibaldi 20, 62100 Macerata, Italy; 2 Department of Human Sciences, University of Verona, Lungadige Porta Vittoria, 17, 37129 Verona, Italy; erika.branchini@univr.it

**Keywords:** creativity, representational change, thinking in opposites, hints and training interventions to reduce fixedness

## Abstract

In this paper, we focus on the link between thinking in opposites and creativity. Thinking in opposites requires an intuitive, productive strategy, which may enhance creativity. Given the importance of creativity for the well-being of individuals and society, finding new ways to enhance it represents a valuable goal in both professional and personal contexts. We discuss the body of evidence that exists concerning the importance of the first representation of the structure of a problem to be solved, which determines the baseline representation and sets limits on the area within which a problem solver will explore. We then review a variety of interventions described in the literature on creativity and insight problem solving that were designed to overcome fixedness and encourage people to move away from stereotypical solutions. Special attention is paid to the research carried out in the context of problem solving, which provides evidence that prompting people to “think in opposites” is beneficial. We suggest that an extended investigation of the effects of this strategy in various types of tasks related to creativity is an interesting line of research to follow. We discuss the rationale supporting this claim and identify specific questions, both theoretical and methodological, for future research to address.

## 1. Introduction

Psychologists have been interested in creativity for quite some time (e.g., Csikszentmihalyi 2013; Sternberg 2006; Weisberg 2006) and the topic still stimulates a great deal of empirical research (1152 papers in the last five years found in PsycArticles, searching for the word creativity in the keywords, abstract or title section). This is not surprising. The creation of new ideas and products is important in a society and being creative gives people a sense of pleasure, self-realization and well-being (e.g., Conner et al. 2018; Han et al. 2019; Ryan and Deci 2001; Shen et al. 2020; To et al. 2012). As a result, it is therefore not unusual that empirical research has extensively focused on the design and testing of interventions that might boost creativity in everyday activities (e.g., Richards 2007, 2010; Silvia et al. 2014, 2017) and in specific professional contexts (e.g., Rasulzada and Dackert 2009; Reinartz and Saffert 2013; Rosso 2014; Zheng et al. 2011). In this paper, we discuss our hypothesis that training people specifically to “think in opposites” might represent an intuitive, productive strategy to enhance creativity in various contexts and domains. There have been sporadic references in the literature, even in the relatively distant past, to the idea that the mental activity involved in processing opposition is related to creativity and problem solving. However, there has been no truly comprehensive investigation of this hypothesis. In this paper, we first describe the motivations for our proposal that a systematic analysis of the effects of a prompt to think in in opposites might be a worthy line of investigation. We then suggest potential questions and topics for a future research agenda. 

In Section 2, our proposal is explained within a more general discussion about the role of constraints in creativity. We focus in particular on the constraints concerning the initial representation of a problem to be solved and on the additional constraints resulting from the instructions given to participants (such as encouragements to “be creative”, examples of solutions to look for or to avoid or explicit strategies to follow to prompt creativity). We assess these constraints in terms of how they may hamper or favor creative solutions. In Section 3, we review what can be considered the harbingers of our proposal—that is, a number of intuitions dating back some time and also relating to more recent experiments with interesting results. In Section 4, we discuss the rationale behind our idea that a systematic investigation of an explicit intervention prompting reasoners to think in opposites in various types of creativity tasks might be promising. As part of the discussion, we describe the mechanism on which this prompt is expected to act. We suggest that thinking in opposites combines exclusionary and focusing constraints (Stokes 2005; Tromp 2022a), which help people to overcome cognitive inertia and to trigger a mental switch that leads to a new way of thinking. The strategy that we propose allows the problem solver to broaden the space in which they search by exploring various different alternatives without the need for additional external stimuli (Briggs and Reinig 2010). In this sense, we contend that the strategy represents a way of helping people to think outside the box by thinking in a more focused manner “inside the box”—or, to put in another way, by reshaping the box (Stacey and Eckert 2010). In the final section (Section 5), we look at a number of specific questions, both theoretical and methodological, in relation to potential future research into this topic.

## 2. The Role of Constraints in Creativity: The Structure of the Problem and Directing the Problem Solver’s Search for More Creative Solutions

Research in the field of psychology into creative thinking has shown that constraints are an integral part of creativity. The role of constraints has been discussed in relation to various aspects and/or phases of the creative process, and the relationship between constraints and creativity has been described from a number of different viewpoints. 

For instance, some authors have described this relationship as an inverted U-shaped function, with too many or too little constraints having various negative effects (e.g., Acar et al. 2019; Biskjaer et al. 2020; Medeiros et al. 2014). Others have suggested that creativity is best analyzed in terms of cycles of exploration and exploitation of constraints (see the Integrated Constraints in Creativity model by Tromp 2022a, 2022b; Tromp and Sternberg 2022). These cycles are modulated depending on the types of creative problems considered (e.g., open-ended or closed-ended), individual differences (some people are more inclined towards flexible constraint exploration, others towards persistent constraint exploitation) and task differences (the novelty of the solution is emphasized in certain contexts, while usefulness is emphasized in others). In any case, within this model, constraints are recognized to be critical at each step of the creative process, from problem finding to problem construction, to problem solving, to the evaluation of creative outcomes. Other types of constraints are examined in the Bounded Ideation Theory (Briggs and Reinig 2010). This theory focuses on the constraints to creativity that are related to the limits on human cognition. These include mental ability, attention, working memory capacities, fatigue, understanding the task and the relationship between personal and social goals. Yet another perspective is that of Gilhooly et al. (2007), who analyze the role of strategies in ideation. From their perspective, coming up with creative responses (for instance, relating to the number of different ways in which an object can be used) entails managing interferences corresponding to the object’s habitual usage, its concrete features and the reasoner’s own previous experience. All of these elements constrain participants’ responses. 

In the subsequent two sub-sections, we look in detail at the constraints that pertain to the initial representation of a problem and those that pertain to the instructions to the task. Both of these considerations support our contention that “thinking in opposites” might be an effective way of boosting creativity (see the arguments given in Section 4). 

### 2.1. The Structure of the Problem

The initial representation of a problem in people’s minds is obviously crucial since it establishes “the box” within which the problem solver will take their initial steps in their search for a solution. Since Duncker’s (1935) and Maier’s (1931) pioneering work, it has been acknowledged that fixedness is the key obstacle to break through in order to be able to find alternative solutions.

Insight problems are an excellent example of how the initial mental representation of a problem can be critical and, as such, they are relevant to any analysis of creativity (see Fine et al. 2019). Indeed, this type of task typically requires people to go beyond the most obvious solutions that come to mind, ones which have been generated by a default representation of the problem. In the literature on insight problem solving, fixation within the initial mental representation of a problem is usually referred to in terms of an impasse and the shift needed to overcome this is known as representational change (e.g., Bilalić et al. 2021; Danek 2018; Öllinger et al. 2014). When a representational change occurs, an alternative solution arises that is often (but not necessarily) accompanied by an “Aha!” experience (Danek and Salvi 2020; Danek et al. 2014, 2020). This alternative solution may or may not be the right one (Danek and Wiley 2017), but the feeling of surprise and pleasure that accompanies it (e.g., Bianchi et al. 2022; Canestrari et al. 2018) indicates that a shift has occurred from an initial, more obvious solution, to a less conventional view of the problem and of the ways to solve it.

If we then go beyond insight problem solving and consider other types of creative tasks, several studies have shown that individuals tend to first generate conventional ideas that are limited to a restricted set of categories and to familiar, obvious information, and only later do the ideas become more original (e.g., Beaty and Silvia 2012; Christensen et al. 1957; George and Wiley 2019; Parnes 1961; Hass 2017). This is known as the serial order effect. Some moderators weaken this effect. For example, the effect is less pronounced for people scoring higher in fluid intelligence (Beaty and Silvia 2012). These people are more creative early on and, thus, display a less pronounced serial order effect (or even no such effect at all). 

The serial order effect has been interpreted as a demonstration that in order to generate creative ideas, reasoners usually need to shift their focus away from any easily accessible ideas linked to their first understanding of the problem—and their initial representation of the space in which to search—and direct their search towards representations that are not default. Within the Bounded Ideation Theory (Briggs and Reinig 2010), the difficulty of this shift is explained in terms of attention boundaries. Concepts related to the initial representation of a problem cause the activation of other closely related concepts (referred to as spreading activation). These allow people to follow a train of thought easily, despite the limits of their working memory, but, at the same time, it becomes more difficult for them to quickly switch to a new line of thought that might be more promising. This fixedness is referred to as cognitive inertia. Thus, the best ideas emerge later because the problem solver needs to overcome their initial cognitive inertia to be able to follow less obvious lines of thought. From a different perspective, within an executive interpretation of divergent thinking (e.g., Gilhooly et al. 2007; Nusbaum and Silvia 2011), later responses that are better are probably the result of the reasoner changing the executive strategy that they initially used to approach the problem. People tend to start by using a simple memory retrieval strategy; then, during the process, they switch to different strategies. Whatever the explanation for this, it is generally agreed that the initial default representation of a problem sets some initial constraints. 

### 2.2. The Role of the Instructions Relating to the Task

The instructions given to participants also have the effect of limiting the space that they work in. Various empirical studies have shown that the originality of the solutions proposed increases when the instructions to the task delimit the boundaries of the space within which to search (e.g., Biskjaer et al. 2020; Caniëls and Rietzschel 2013; Chaffois et al. 2015; Medeiros et al. 2014; Moreau and Dahl 2005; Rietzschel et al. 2014; Rosso 2014; Sellier and Dahl 2011; Stokes 2005). Thus, constraints are not necessarily detrimental to creative performance. Indeed, they define the “box” to work in, but, at the same time, they drive the reasoner towards various possible exits, some of which are particularly creative. Leaving a wider, more open space to work in does not necessarily help the reasoner to see the best direction to take. 

In this section, we concentrate on two types of constraints that are relevant to the main point developed in this paper, and both are related to the type of instructions provided. One concerns instructions that include examples of solutions given to the participants before they start to generate their own ideas. In this way, the reasoner has to take into account not only the structure of the problem but also the kind of solution to look for, as suggested by the example. Providing examples beforehand can at times lead to negative outcomes (e.g., Agogué et al. 2014; Smith et al. 1993). In fact, participants who are exposed to examples beforehand tend to add constraints based on the examples they have just seen, which are not necessarily relevant to the problem that they are trying to solve. This limits both the number and the quality of the potential solutions generated (Chrysikou and Weisberg 2005; George and Wiley 2020, experiment 2; Jansson and Smith 1991; Purcell and Gero 1996; Smith et al. 1993). Similar negative effects have also been observed in group brainstorming conditions (Kohn and Smith 2011). Giving prior examples, however, is not always negative. If the examples are innovative, the fixation effect may play a positive role in terms of the generation of new ideas (Agogué et al. 2014; George et al. 2019; Sio et al. 2015; Yagolkovskiy and Kharkhurin 2016). Furthermore, giving non-innovative examples of solutions together with an explicit instruction to *avoid* this kind of solution is another way of restricting the exploration space in a fruitful way. Suggesting which paths to avoid has turned out, in some cases, to be even more productive than suggesting which path to follow (Chaffois et al. 2015; George and Wiley 2020, experiment 1; Shin et al. 2020). 

A second constraint related to instructions concerns the effect of an explicit instruction to “be creative”. For many years, originality and quality have been considered to be by-products of quantity (Osborn 1963). Indeed, the majority of studies on creativity have instructed people to “think of as many ideas or responses as possible”. The assumption is that the fact that there are many responses increases the probability that there will also be novel, original ideas (Simonton 2010). Solid empirical evidence has, however, shown that an increase in the number of ideas does not significantly increase the number of good ideas produced (e.g., Baruah and Paulus 2008; Reinig and Briggs 2008). Conversely, quality improves when participants are instructed to “be creative” (e.g., Acar et al. 2020; Harrington 1975; Forthmann et al. 2016; Nusbaum et al. 2014; Runco et al. 2005; Said-Metwaly et al. 2020; Wilken et al. 2020). This instruction orients individuals’ attention away from common ideas and towards more original ones (Said-Metwaly et al. 2020). The quality is further improved when a prompt to “be creative” is accompanied by advice on certain strategies to adopt if the participants get stuck, such as, for example, telling them to consider objects of similar shapes, using the same object more than once or disassembling and reassembling the object’s parts (Nusbaum and Silvia 2011; Wilken et al. 2020). All of these instructions direct the participant towards the kind of solution to look for and the strategy to follow and, in this sense, they add limitations. At the same time, however, they are beneficial to the quality of the solutions.

### 2.3. Hints and Training as Aids to Overcoming Fixation 

Hints and training represent a third aid that psychologists have studied in order to help reasoners to overcome an initially unsuccessful approach and stimulate them to think along alternative lines. These techniques have been implemented in particular in relation to insight problems. Along the continuum that goes from open-ended to closed-ended creativity problems (see Briggs and Reinig 2010; Tromp 2022b), insight problems are usually considered to be closed-ended problems since they have only one correct solution. According to Tromp (2022b), open-ended problems are more likely to be characterized by a sequence of explorations followed by an exploitation of the constraints that have arisen during the sequence of explorations. In contrast, closed-ended problems usually involve a sequence that starts with the exploitation of the constraints inherent to the problem and this leads to a blockage, which is then followed by an exploration of alternate representations of the problem. Hints and training are designed to help reasoners to overcome blockages. The difference between hints and training corresponds, broadly speaking, to the distinction between automatic, unconscious, associative processes versus controlled, conscious, analytical processes (Evans and Stanovich 2013a, 2013b). However, Bowden (1997) distinguished between reportable hints, unreportable hints and undetectable hints, which demonstrates that hints too might involve various levels of awareness. 

In some studies, improvements in performance were achieved when the experimenters hinted implicitly at the critical feature by manipulating the representation of the problem in such a way that it would be noticed straightaway (e.g., Broderbauer et al. 2013; Dow and Mayer 2004; Hattori et al. 2013; Murray and Byrne 2013). In other studies, improvements were achieved when the participants were presented with an explicit procedure to be consciously followed in an analytical way (e.g., Patrick and Ahmed 2014; Patrick et al. 2015; Walinga et al. 2011; see also the experiments by Wilken et al. 2020 involving an instruction to “be creative” followed by suggestions regarding a strategy to adopt in case there is a blockage). 

Among the various types of hints and training that have turned out to be beneficial to problem solvers are a subset of interventions in which opposition is a key factor in terms of helping the participant to identify the critical dimension to work on. These will be reviewed in detail in the next section since they are important premises with regard to our proposal to extend the exploration of the role of “thinking in opposites” in creative tasks beyond the research carried out thus far. 

## 3. Thinking in Opposites and Creativity: Traces of a Link

Even though there has not yet been a systematic investigation of the hypothesis that thinking in opposites facilitates creativity, there have been isolated references in the literature to a relationship between these two mental activities. Traces of this connection can be found, for instance, in a number of biographical and autobiographical sources, as well as in some interview studies with scientists and artists (Rothenberg 1983). Opposition emerged as a key factor in various phases of their creative processes. Moreover, in word association tasks, it was found that participants who were more creative produced more opposite words, and with lower latency, as compared to less creative participants (Carroll et al. 1962; Rothenberg 1973a, 1973b; Wynne et al. 1964). In the work of Rothenberg (1973a, 1973b), for example, the mean percentage of responses to stimuli evoking opposites (i.e., adjectives, nouns or verbs that tend to evoke opposites for the majority of people in free association tasks, e.g., dark–light, soft–hard, man–woman, sickness–health) was significantly higher for the participants in the high-creativity group as compared to the low-creativity group. These two groups were defined based on self-rating, family ratings and educational ratings. A difference between the two groups also emerged in terms of their response speeds to stimuli evoking opposites. Both of these differences in performance were specifically related to the task involving stimuli evoking opposites; they were not found when stimuli that did not evoke opposites were used. Furthermore, Dumas and Schmidt (2015) observed that a greater capacity to conceive of and use various types of oppositional reasoning (namely anomaly, antinomy and antithesis), as measured by the TORR test (Dumas et al. 2013), predicts more creative behavior.

Many techniques based on opposition have been suggested at various points in time in order to facilitate creative thinking. For example, De Bono (1967) advised reasoners not to stop at the first potential solution, but to keep exploring alternative solutions by deliberately reversing the elements of the problem and the relationship between them. Zingales (1974) and Levine (1988) pointed out that a profitable way to think creatively in problem solving consists of stretching a situation or its individual features up to extreme values using strategies that presuppose a variety of oppositional processes (e.g., exaggeration and diminution, enlargement and dissection or addition and subtraction) or choosing one of these strategies and then transforming the results obtained into its opposite. Opposites are also implied in the Contradiction Analysis and Contradiction Matrix used in the TRIZ (a Russian acronym that translates into English as the “theory of inventive problem solving”). The TRIZ is a model-based technique that was developed in 1946 (see Altshuller 1999). It is still used today to generate innovative solutions relating to technical systems, mostly in industry and management. It is based on the idea (based on an analysis of hundreds of thousands of inventions in a variety of different fields) that there are generalizable patterns in the nature and characteristics of problematic situations (including the presence of a dilemma between contradictory elements) and that these can be used to solve new problems. Two types of contradictions are considered. Technical contradiction occurs when there are alternatives that will improve one parameter of a system, but this is at the expense of another parameter, which worsens. Physical contradiction occurs when the parameters of a product or service must have two opposite states (e.g., the product needs to be simultaneously hot and cold, or both large and small). According to the TRIZ, understanding how to structure a problem in terms of contradictions is an essential step, since once the contradictions are identified, they can be resolved by referring to analogies with similar, previously solved problems.

Traces of a relationship between opposites and problem solving are also present in early texts dealing with insight problem solving. Duncker (1935) commented that the transformation needed to overcome fixedness is sometimes a shift from the original function to a contrary function. To explain his idea, he referred to a hammer and anvil, a father and his son and a radius and a tangent—that is, elements with different functions but linked to each other in some way, as in adjectives such as long and short, to cite his example. The idea that contrast is critical to insight problem solving is also implied in Wertheimer’s ([1920] 1945) definition that the reorganization of the phenomenal structure of a problem needed in order to find the solution consists of dividing what appears to be a unit and unifying what appears to be separate. An analysis of many classic visuo-spatial insight problems reveals that, in effect, the re-structuring required to overcome an initial representation of a problem that is unproductive implies transforming one or more spatial aspects of the problem into their opposites (see Branchini et al. 2015b; Bianchi et al. 2020). For example, in the ping-pong ball problem (Ansburg and Dominowski 2000), the aim is to work out how to throw a ping-pong ball so that it will travel a short distance, come to a dead stop and then move back on its tracks, without bouncing it off any surface or tying it to anything. The mental model that initially comes to mind contains an implicit assumption of a horizontal trajectory (Murray and Byrne 2013). In order to solve the problem, however, it is necessary to imagine the ball following a vertical trajectory. The same holds for Maier’s nine dots problem, where the implicit constraints introduced by default in problem solvers’ minds leads them to only trace lines inside the square formed by the configuration, rather than also considering using the space outside its amodal boundaries. Moreover, problem solvers tend to assume that the lines will necessarily be horizontal or vertical (according to the visual organization of the nine dots) and thus oblique lines are initially disregarded. Likewise, in Katona’s five squares matchstick problem, for which participants are asked to reduce the number of squares from five to four by moving only three matches, the tendency is to move three external matches that are visually segregated, whereas the solution requires them to move three interior matches (which are visibly all connected to one another), and this is where the difficulty lies. 

In the case of visuo-spatial insight problems, giving hints or training people to think in opposites seems to be of some help. In one study (Branchini et al. 2015a), the participants were invited to list all the spatial properties relating to the structure of the problem and identify their corresponding opposites before embarking on their search for the solution. This was a hint since the participants had not been told why this strategy might help. In another study (Branchini et al. 2016), thinking in opposites was proposed as an explicit strategy: the participants were given training that showed them how their task would be easier if they systematically tested whether transforming one aspect of the initial representation into its opposite would lead to the solution. In this case, an explicit instruction and examples were used. In a third study (Bianchi et al. 2020), a comparison was made between two training conditions and two hint conditions, all of which were based on opposites. In all of these studies, in both training and hint conditions, a prompt to explore the structure of the problem in terms of opposites led more frequently to greater success rates as compared to the control groups. It also had the effect of modifying the participants’ approach to the problem. An analysis of the verbal interactions within the groups in the hint condition in Branchini et al.’s (2015a) study revealed that the participants were more focused on a careful analysis of the perceptual structure of the problem and on performing perceptual operations on the elements of the figure with a view to finding perceptual solutions, rather than referring to previously learnt notions and formulas. An analysis of the drawings done by the participants in the training condition tested in Branchini et al. (2016) showed that they manifested less fixedness and expanded their search space bidirectionally (i.e., imagining separating something that was unified and unifying something that was separated). The process that they used was also more effective since they concentrated on properties that were, in fact, relevant to the solution. A comparison between hint and training conditions (Bianchi et al. 2020) suggests that thinking in opposites is more beneficial when it is performed on an explicit level. Success rates were better when participants were trained to use this strategy or the hint that they were given in any case involved a certain amount of overt, conscious work. 

## 4. The Rationale for Setting Up a More Extensive Research Program on Opposites and Creativity

The studies referred to in the previous section form only a partial picture. Systematic testing of the hypothesis that an explicit prompt to think in opposites might benefit creativity would need a great deal of further investigation. Before listing the methodological and theoretical questions that a future research agenda might need to address (Section 5), let us focus on the rationale for taking this hypothesis seriously. Why might the considerations reviewed in the previous sections arouse researchers’ curiosity regarding whether thinking in opposites may be useful and may lead to the ideation of more creative solutions in a variety of contexts? 

A first consideration is that this technique is within everyone’s reach. We all possess an intuitive idea of what opposites are. It is a primary intuition that has its roots in infants’ pre-verbal categorization (e.g., Casasola 2008; Casasola et al. 2003). Opposites and dimensions are key structures in the human perceptual experience of space (Bianchi et al. 2011; Bianchi and Savardi 2018) and in conceptual spaces (Gärdenfors 2014). They are pervasive in natural human languages (e.g., Croft and Cruse 2004; Jones 2002, 2007; Murphy 2003). Both children and adults have an intuitive idea of what two opposite properties (Bianchi et al. 2011, 2017a, 2021b), two opposite figures (Bianchi and Savardi 2008), two opposite sounds (Bianchi et al. 2017c; Biassoni 2009), two opposite postures (Bianchi et al. 2014) or two opposite transformations might be (Bianchi et al. 2021a; Capitani et al. 2020). Moreover, people intuitively use opposites to help them to find alternatives to what really happens in everyday life (i.e., counterfactual thinking), or in order to justify, for instance, a negative performance (Byrne 2018; Epstude and Roese 2008; Sirois et al. 2010; Tyser et al. 2012). This is a primal ability that even preschool children already possess (e.g., Beck and Guthrie 2011; FitzGibbon et al. 2019; Rafetseder et al. 2010). We all easily understand what the term “opposite” means and thus a prompt to think in opposites can be given to both young people and adults, and in professional as well as non-professional contexts. 

A second consideration is that, although a prompt to “think in a different way” or “think outside the box” is compelling, telling someone to “think in opposites” might act on a different level. As revised in Section 2.2, an instruction to “be creative” works better when it is accompanied by advice on what type of strategy to follow (e.g., Forthmann et al. 2019; Nusbaum and Silvia 2011; Wilken et al. 2020). According to the Integrated Constraints in Creativity model, creative outcomes are the result of the successful leveraging of constraints that can be functionally divided into exclusionary constraints and focusing constraints (Tromp 2022a, 2022b). These ideas are useful in terms of elucidating why thinking in opposites might be beneficial to creative performance. Exclusionary constraints direct the search away from something (i.e., avoid x), but not towards anything in particular. This kind of constraint is involved when participants are provided with examples of the type of solution to avoid, or they are advised to avoid the types of obvious ideas that other people might also come up with. In contrast, focusing constraints directs the search for a creative outcome toward a specific concept (use y) or category (search within y). This kind of constraint is involved when examples of desirable solutions are suggested, or people are explicitly told which driving strategies to follow in their search phase. Thinking in opposites combines both of these constraint types. Opposites, by definition, consist of pairs of properties. By systematically analyzing the properties of the initial representation and turning them into their opposites, the reasoners are able to see the constraints but, at the same time, see the exact direction that they need to take in order to overcome these constraints. In other words, one of the two poles forming an opposite pair will reveal the tacit barrier or assumption that is embedded in the initial representation of the problem and is to be avoided. The other pole will offer a potential alternative. 

Gilhooly et al. (2007) suggested that generating creative responses entails managing interference from several sources (from the concrete aspects of an object or representation to its more accessible uses and functions in addition to the participant’s previous responses) and identifying strategies in order to do this. Thinking in opposites would seem to represent a straightforward candidate. It prompts reasoners to focus systematically on all of the features that define the problem set (thus, its most evident, concrete, functional aspects will also be noticed), and then to create alternative solutions by reversing each of these. In this way, the strategy also reduces rumination over the same ideas and previous responses and rather encourages continuous switching of the direction in which to search (see the discussion relating to cognitive inertia and switching in Section 2.1). 

Furthermore, using opposites as a strategy has the effect of extending the area in which to search for a solution, but it does this in a restricted way since it is bounded by precise dimensions. This combination of openness and boundaries has been acknowledged to be an important requisite for an effective cognitive heuristic in insight problem solving (e.g., Öllinger et al. 2013). It is also in agreement with the literature reviewed in Section 2.2, which demonstrates the benefits of devising instructions that delimit the boundaries of the space to explore by providing examples to be followed or avoided, as compared to not providing anything at all.

Some attempts have been made to expose participants to ambiguous figures (such as the duck-rabbit or the Necker cube) prior to a creative task in order to encourage them to consider multiple representations of the situation (e.g., Doherty and Mair 2012; Laukkonen and Tangen 2017; Wiseman et al. 2011; Wu et al. 2016). Ambiguous figures in fact involve a reversal from an initial, apparently univocal, interpretation of a figure and reveal a novel representation of the same figure. This reversal suggests multiple representations. This is exactly what is required in creative tasks. One of the explanations in the literature on this subject regarding why re-interpreting perceptual stimuli should prompt a re-interpretation of conceptual stimuli also supports our expectation that thinking in opposites might work. It has in fact been stated by Alter et al. (2007), Kan et al. (2013) and Stoycheva (2010) that any conflict between competing representations creates an opportunity to step aside from the default, premature response and deliberately engage in a more analytical mode of thinking based on control processes that are important in creative thinking. Creative thinking does indeed involve a complex series of processes, including both implicit associative processes and explicit control processes (Beaty et al. 2016). Explicit control processes (e.g., control over goal setting, planning, deliberate shifts in attentional focus and intentional mind wandering) are important in various phases of creative thinking, from gathering and structuring information, to ideation and verification of the effects of an idea (e.g., Corazza and Agnoli 2015; Feldhusen 1995; Vannucci and Agnoli 2019). Thinking in opposites operates on this level. In one way, reversing the individual characteristics of an idea or representation into their opposites involves a process that is similar to that involved in the duck-rabbit or the Necker cube conundrums. However, in these conundrums, the process is mostly unconscious and automatic. Conversely, when one deliberately thinks in opposites, reversing the characteristics of the initial idea or representation is a conscious strategy, however simple. It involves explicit control processes (such as the identification of the informative elements and the ideation of a hypothesis or potential transformation), but, at the same time, it is based on implicit, associative processes (i.e., the pairing of opposites).

Moreover, the prompt to think in opposites is not based on a content-related suggestion. Most of the suggestions provided to participants in insight problem solving tasks in order to help them to overcome fixedness are specific to the content of the problem. For instance, in a study involving Katona’s (1940) matchstick problem, the final shape that the matches should have was embedded in the form of a logo printed on the response sheet given to participants (Broderbauer et al. 2013). In other cases, the solution was presented subliminally (Hattori et al. 2013) or the wording of the problem was changed to downsize the importance of the elements that were usually the cause of the impasse (Murray and Byrne 2013). This is often a good method to use, but it is relatively complex since there need to be as many different hints as there are individual features of the problem. A prompt to think in opposites does not make an explicit reference to any individual feature; reasoners must identify the features each time, based on the problem at hand. The prompt is general, and therefore it has a wide range of application.

## 5. Methodological and Theoretical Questions for a Future Research Agenda

There is potentially a great deal of research to be implemented from here on in order to design new interventions aimed at stimulating thinking in opposites in relation to various types of creative tasks, and in order to assess the efficacy of these interventions. 

Among the issues that need to be addressed regarding this research agenda is a set of questions concerning the indexes to be used to assess the efficacy of a prompt to think in opposites on creative performance. One might want to investigate whether this impacts the number of solutions that reasoners are able to find (fluency), as well as assess the novelty and quality of the solutions or their usefulness (Martin and Wilson 2017; Runco and Jaeger 2012). The few studies that have explored the effect of a hint or training to think in opposites in relation to visuo-spatial insight problem solving (Branchini et al. 2015a, 2016; Bianchi et al. 2020) did not find evidence of an effect on fluency, but did reveal an effect on the quality of the solutions. These results, however, are limited to insight problem solving tasks, which are closed-ended problems (Tromp 2022a, 2022b), and, even more specifically, to visuo-spatial insight problems. If this type of training is applied to other types of creativity tasks, one would also need to reconsider which indexes are the most informative with reference to the creativity of a response. This is a general issue concerning creativity studies (for a systematic literature review of the advantages and weaknesses of various types of measurements of creativity, see (Said-Metwaly et al. 2017)). Classic divergent thinking tests are reasonably reliable and are also predictive of creative performance according to certain studies (Beketayev and Runco 2016; Cramond et al. 2005; Runco and Acar 2012; Runco et al. 2010), although they suffer from the confounding effect of fluency (e.g., Forthmann et al. 2020) and reliability issues (e.g., Forthmann et al. 2021) according to other studies.

It would also be interesting to measure the effect of training by means of self-ratings of creativity (Bergum and Bergum 1979). The recent literature on insight problem solving and creativity has begun to pay more attention to the phenomenal or meta-cognitive experience of the reasoner engaged in the task (e.g., Ammalainen and Moroshkina 2021, 2022; Danek et al. 2014, 2020; Danek and Wiley 2017; Kaufman et al. 2016; Laukkonen and Tangen 2018; Moroshkina et al. 2022; Pétervári and Danek 2019; Shen et al. 2016; Spiridonov et al. 2021). It would thus be worth exploring whether being prompted to think in opposites is perceived by the reasoners themselves as useful and stimulating, as well as indicating how this impacts their motivation. This latter aspect is another crucial factor since motivation moderates the effect of interventions aimed at prompting creativity, both in terms of fluency and flexibility (Said-Metwaly et al. 2020).

Another aspect to take into account concerns time limits. Some studies have reported that participants in creativity tasks had higher scores when they were given more time (e.g., Johns and Morse 1997; Roskes et al. 2013), whereas others have reported contradictory results (e.g., Acar et al. 2020; Johns et al. 2000; Khatena 1972; Morse et al. 2001; Sajjadi-Bafhgi 1986; Sajjadi-Bafhgi and Khatena 1985). Said-Metwaly et al. (2020) found that longer time limits enhanced the scores relating to originality, but no convincing evidence was found of an effect on fluency or flexibility scores. Paek et al. (2021) found that people’s performance in divergent thinking tasks improved when they were given more time (especially in terms of fluency and flexibility, but not so much in terms of originality). However, they found an inverted J-shaped relationship—that is, the performance improved as more time was allowed, but this improvement slowed down progressively over time. In other words, a sufficiently long time period contributes to maximal performance, but excessive time does not necessarily enhance it proportionately and untimed conditions are not as good as timed conditions with sufficient time. The authors interpret these findings as evidence that providing ample time may encourage people to follow their own pace during the ideation process, which will eventually lead to a more creative performance. However, at the same time, the awareness that the time allotted is not infinite would make them more efficient.

It would be worth analyzing the effects of training people to think in opposites with both shorter and longer time limits and in relation to whether the task is open-ended or closed-ended. Since the order of the solution exploration phase and of the constraints’ exploitation phase is different in open-ended and closed-ended tasks (Tromp 2022a, 2022b), the benefits of training might emerge early on in the ideation process for one type of task and later for the other type of task. The ideation function described by the Bounded Ideation Theory (Briggs and Reinig 2010) would be a suitable frame of reference to model the effect of training in terms of time. Indeed, this function describes any variability occurring in the ratio of good ideas (i.e., quality) to the total number of ideas (i.e., quantity) in time during ideation. A curve is created by plotting the cumulative number of ideas on the *x*-axis and the cumulative number of good ideas on the *y*-axis. The shape of the function would show whether training had affected the ideation curve and at what point.

The distinction between creativity tasks that use figures and shapes and those that are verbal might also be relevant and needs to be considered in future research. Said-Matwaly et al. (2020) found that the benefits of using different types of instructions varies with these two types of task. Plucker (1999) and Ulger (2015) found only weak correlations between performance relating to verbal divergent thinking as compared to figural divergent thinking. Moreover, creativity tasks using figures and shapes seem to be subject to more task-specific variance than verbal tasks (Chen et al. 2005; Palmiero et al. 2010). The effect of explicit instructions on creative behavior, for instance, differed for tasks that involved drawing abstract shapes and figurative shapes (e.g., Forthmann et al. 2019). Similarly, the effects of showing participants ambiguous shapes prior to performing a creativity task vary depending on the task. The benefits are not as clear-cut for verbal insight problems (e.g., Olszewska and Sobkow 2021), but positive effects were found in a task involving the creation of stories consisting of short sentences based on three unrelated words (Wu et al. 2016, exp. 2). Riquelme (2002) found a significant relationship between the ease with which people detected ambiguous figures and their performance in a visuo-spatial creative synthesis task. Prior exposure to ambiguous figures improved performance in Alternative Uses tasks (Guilford et al. 1978), in which participants are asked to list as many possible different uses for common objects (Wiseman et al. 2011, esp. 2; Wu et al. 2016, exp 1), and also in the Pattern Meanings Test (by Wallach and Kogan 1965), in which participants are requested to identify as many meanings as possible relating to simple abstract patterns (Olteţeanu et al. 2019). Based on the results of these studies, a comprehensive exploration of the effects of prompts to think in opposites on both figurative and verbal tasks is therefore worth carrying out. To date, we know that there are some beneficial effects related to thinking in opposites in insight problem solving settings involving visuo-spatial problems (see, in Section 3, references to Branchini et al. 2015a, 2016; Bianchi et al. 2020), and that evidence of a link between creativity and the ease with which the participants manipulated opposites was also found in a word association task (see, in Section 3, Rothenberg 1973a, 1973b). However, we do not have either directly comparable results or a systematic, separate investigation of the effect of thinking in opposites in various types of verbal tasks as compared to figurative tasks. 

Other potential variables with a moderating influence relate to the individual ability of participants (e.g., Said-Metwaly et al. 2020). For instance, Khatena (1972, 1973) found that the effect of time limits on creative responses was affected by the participants’ level of creativity. Higher levels of fluid intelligence were associated with the generation of more original ideas (Nusbaum and Silvia 2011; Miroshnik and Shcherbakova 2019) and these participants profited more from an instruction to be creative, as well as from an instruction to be creative plus advice on a strategy to follow (Forthmann et al. 2019; Nusbaum et al. 2014). Divergent thinking and performance in terms of creativity also turned out to be modulated by gender effects (for a review see Baer and Kaufman 2008) and by the openness to experience personality trait (Dollinger et al. 2004; Feist 1998; Hughes et al. 2013; McCrae 1987). It cannot be excluded that the effect of a suggestion to think in opposites may be modulated by similar individual variables (partial evidence of this emerged in Bianchi et al. 2017b).

Finally, another question to address concerns the efficacy of thinking in opposites in individual as compared to group conditions. Indeed, in the studies reviewed in Section 3 with reference to insight problems (Bianchi et al. 2020; Branchini et al. 2015a, 2016), hints and training to use opposites were only effective in small group settings. If one starts from Poletiek’s (1996) observation that it is always easier to contradict another person’s best guess than it is to question one’s own, one can see why it might be easier to “think in an opposite way” in a group condition (see also Augustinova 2008). After the others in the group have made their suggestions, it is relatively easy to simply try the reverse. However, there is also evidence of a relationship between the processing of opposites and creativity in individual settings. For instance, an association between opposite word association and creativity has been found with people engaged in individual tasks (Rothenberg 1973a, 1973b). The same holds for the results of the studies reviewed in Section 4 concerning the benefits of stimulating flexibility by means of ambiguous figures. It would be interesting to ascertain the benefits of and limits to the application of this strategy in both group and individual conditions. This represents another area of research to be explored. 
